# Metabolic characteristics of ischaemic preconditioning induced performance improvement in Taekwondo athletes using LC‒MS/MS-based plasma metabolomics

**DOI:** 10.1038/s41598-024-76045-1

**Published:** 2024-10-19

**Authors:** Ziyue Ou, Liang Yang, Jingyun Wu, Mingxin Xu, Xiquan Weng, Guoqin Xu

**Affiliations:** 1 College of Martial Arts, Guangzhou Sport University, Guangzhou, 510500 China; 2 Department of Physical Education, Sun Yat-Sen University, Guangzhou, 510275 China; 3 The Fifth College of Clinical Medicine, Guangzhou University of Chinese Medicine, Guangzhou, 510405 China; 4 College of Exercise and Health, Guangzhou Sport University, Guangzhou, 510500 China; 5 Guangdong Provincial Key Laboratory of Physical Activity and Health Promotion, Guangzhou Sport University, Guangzhou, China

**Keywords:** Metabolic characteristics, Ischaemic preconditioning, Sports performance, LC‒MS/MS-based plasma metabolomics, Taekwondo athletes, Biochemistry, Metabolomics

## Abstract

In recent years, ischemic preconditioning (IPC) has garnered significant attention in sports research. While IPC has demonstrated positive effects in high-intensity sports such as judo and swimming, its potential benefits for enhancing the performance of Taekwondo athletes have not been extensively studied. This study aimed to investigate the effects of IPC on taekwondo performance and to observe the metabolic characteristics associated with enhancing sports performance via LC‒MS/MS-based plasma metabolomics. Seventeen participants underwent the repeated frequency speed of kick test (FSKT) after IPC, along with pre- and post-exercise plasma metabolite analysis. Differential abundance metabolite analysis, enriched pathway analysis, and weighted gene coexpression network analysis (WGNCA) were employed to delve into metabolic characteristics. The findings highlighted a significant enhancement in FSKT performance in the experimental group. Metabolomic analysis revealed 109 differentially abundant metabolites, including Dl-lactate, hypoxanthine, acetylcarnitine, and acetylsalicylic acid. Enriched pathway analysis revealed pathways such as pentose and glucuronic acid interconversion, ascorbic acid and aldonic acid metabolism, the pentose phosphate pathway (PPP), and the Warburg effect. In conclusion, IPC can significantly increase the specific athletic abilities of Taekwondo athletes, with enhancements linked to anaerobic metabolism, PPP utilization, the Warburg effect for energy production, redox system stability, reduced muscle fatigue, and pain alleviation.

## Introduction

IPC was first discovered by MURRY and has been shown to have significant effects on protecting tissues from long-term ischemic damage, particularly myocardial ischemic damage^1^. Recent studies have demonstrated that IPC can enhance the athletic performance of athletes in various sports. For example, IPC was shown to increase athletes’ maximum oxygen uptake by 3% in incremental load power bicycle tests^2^. Additionally, in studies using constant maximum load bicycle tests, IPC was found to reduce subjective fatigue, prolong the time to exhaustion, and increase maximum oxygen uptake^3^. These findings highlight the positive impact of IPC on aerobic exercise capacity. Furthermore, research has indicated that IPC can also improve anaerobic endurance performance. In the Wingate anaerobic power test, IPC was found to increase the average output power of participants in lower limb tests^4^. Similar results were observed in basketball athletes subjected to the same test^5^. Notably, IPC has been shown to be beneficial for enhancing the performance of judo athletes in swimming^6,7^. However, conflicting studies have suggested that IPC may not universally enhance exercise performance. Some research has indicated that IPC does not improve performance in repeated sprints during all-out sprint phases^8^. Cyclists did not experience improvements in aerobic capacity or 4-km racing performance after IPC interventions^9^. Moreover, while IPC has been found to enhance swimmers’ 100-m performance^6^, it may not have the same effect on athletes competing in 200-m speed swimming races^10^. Therefore, further investigation into the mechanisms underlying the effects of IPC on sports performance is crucial for its optimal application in training regimens. Notably, the impact of IPC on Taekwondo performance remains unexplored.

Research indicates that IPC can produce various biologically active substances, such as adenosine, NO, catecholamines, and opioids^11^. IPC is known to mitigate ischemia‒reperfusion injury by acting on selective mitochondrial ATP-sensitive potassium channels (mK_ATP_) and safeguarding tissues from ischemia-induced damage. These mechanisms play crucial roles in modulating vascular tone and facilitating muscle contraction^2,12–14^. Notably, the mode of action of IPC involves regulating energy metabolism and combating oxidative stress. By enhancing the mitochondrial uptake of acetyl-CoA (a glycolysis byproduct), IPC helps maintain lactate levels within a metabolically suitable range, which is vital for enhancing anaerobic exercise performance^15,16^. Furthermore, IPC can increase mK_ATP_ and adenosine levels, leading to improved vasodilation, increased muscle blood supply, and increased oxygen transport efficiency^2^. Some studies have suggested that IPC can increase muscle fatigue resistance^17^, potentially contributing to delayed exhaustion. While there is abundant research on the protective mechanisms of IPC, there remains a need for a more comprehensive and thorough investigation into how IPC enhances exercise performance.

Metabolomics has made significant advancements in understanding metabolic changes and identifying potential biomarkers in recent years. Nontargeted metabolomics technology provides a comprehensive view of various metabolites in samples^18^. A recent study conducted a 10-day experiment in which IPC was applied to subjects and utilized metabolomics to establish a connection between IPC and cholesterol metabolism^19^. However, the use of metabolomics in acute IPC, especially in sports research, remains limited. This study aimed to investigate the effects of acute IPC on the performance of taekwondo athletes and the metabolic characteristics associated with enhanced sports performance via LC‒MS/MS-based metabolomics. The goal of this research is to provide scientific insights into the application of IPC in taekwondo training and competitions and to lay the groundwork for utilizing IPC to improve sports performance.

## Methods

### Subjects

Seventeen adult male Taekwondo athletes participated in the study. The exclusion criteria included acute or chronic diseases such as anxiety, depression, cardiovascular system diseases, sports, and metabolic diseases, as well as creatine supplementation, alcohol or caffeine intake within 24 h prior to the experiment, and strenuous physical activity within the same timeframe. The inclusion criteria were adult males over 18 years old, Taekwondo professional athletes with a national level of two or more athletes, and those who had maintained regular training in the past three months.

All participants were fully informed of the experimental process and provided signed informed consent. The research protocol received approval from the Ethics Review Committee of Guangzhou Institute of Physical Education (ID Number: 2023LCL-81) and adhered to the guidelines of the Declaration of Helsinki.

### Experimental protocol of the study

The experiment utilized a self-parallel paired design in which participants were tasked with completing two experiments, one under 220 mmHg pressure (experimental group) and the other under 20 mmHg pressure (control group), with a minimum of one week between the two experiments. The order in which the participants were subjected to the experimental and control conditions was randomized.

**Fig. 1:**
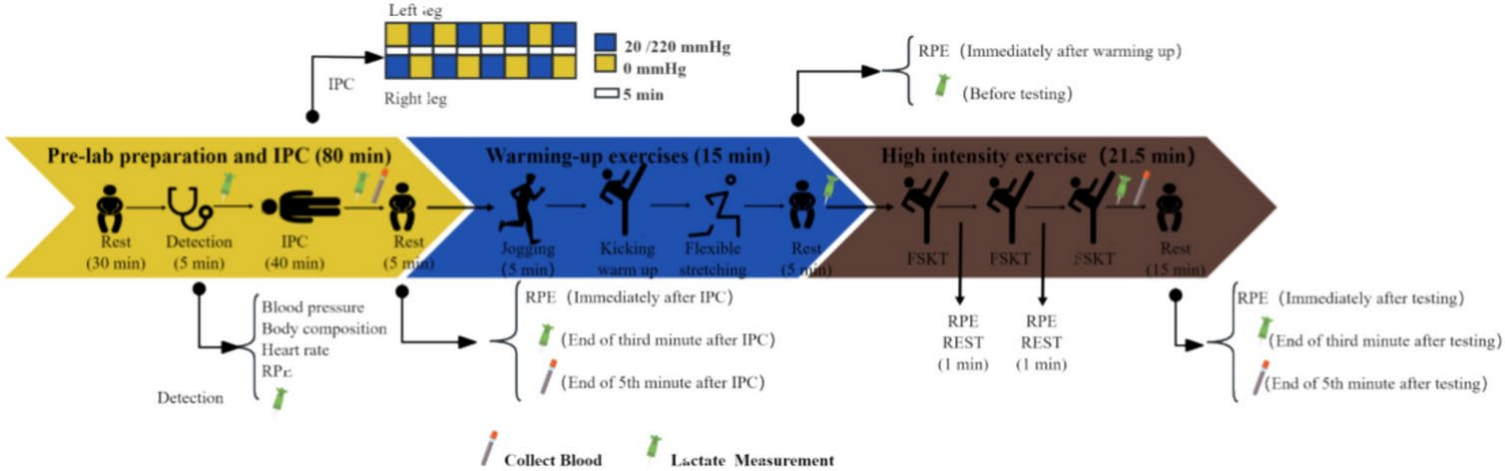
Experimental flowchart.

Upon arrival at the test site, the subjects rested quietly for 30 min before their blood pressure was monitored. They then underwent a body composition test, wore a heart rate monitor, reported their rating of perceived exertion (RPE), and had their blood lactate levels measured from fingertip blood samples. Following this, a 40-min compressive intervention was administered, with an immediate RPE test upon completion, a blood lactate test 3 min later, and blood collection from the median cubital vein via an EDTA-K2 anticoagulant tube 5 min later. The warm-up activities consisted of 10 min of jogging, kicking, and other exercises, followed by flexibility and stretching exercises. RPE was assessed after the warm-up, followed by a period of rest and finger prick blood sampling. The Taekwondo-specific test involved three sets of FSKT tests with a 1-min rest interval, with the RPE recorded between sets. After the test, the RPE, blood lactate levels, and blood samples were collected, and the heart rate was monitored for 15 min after exercise. The experimental process is illustrated in Fig. 1.

In addition, only 8 randomly selected athletes in the experimental group underwent venous blood collection and metabolomic analysis.

### Basic *indicator* measurement methods

Heart rate was monitored via a heart rate monitoring device (Polar H10; Finland). Blood pressure was measured via an oscillometric method^20^ with a blood pressure monitor (Omron, HEM-1020). Body composition analysis was performed via an InBody 370 body composition analyser (Korea). Blood samples were obtained via finger prick tests, and blood lactate concentrations were analysed via a Biosen C-line analyser (Biosen C-line, EKF Diagnostics). Fatigue levels were subjectively assessed through the Rating of Perceived Exertion (RPE)^21^.

### IPC

Participants underwent IPC intervention while in the supine position and breathing normal oxygen. A blood pressure cuff was positioned on the middle and upper thirds of both thighs, with the right leg cuff inflated to 220 mmHg and the left leg cuff at 0 mmHg for 5 min. Subsequently, the right leg cuff was deflated to 0 mmHg, while the left leg cuff was raised to 220 mmHg for another 5 min, completing one cycle^5,22,23^. This cycle was iterated 4 times, totaling 40 min.

### Taekwondo performance testing protocol

The FSKT is a method used to evaluate the specific sports performance of Taekwondo athletes^24–26^. In this study, participants were instructed to complete three sets of tests, with a 1-min break between sets, mimicking the structure of a formal Taekwondo competition. During each set of tests, participants were asked to use kicking scoring equipment to kick at full speed with both legs, which was continuously striking for 10 s followed by a 10-s rest. This pattern was repeated for 5 rounds of kicking and 4 rounds of rest, totaling 90 s. After each set, there was a 1-min break before the next set began. The number of kicks was recorded via the World Taekwondo Grand Slam competition electronic protective gear (professional version, China) and the corresponding competition system. The total number of kicks per round (10 s), the total number of kicks in five rounds (90 s), and the Kick Decrement Index (KDI) were used to assess the participants’ sports performance. Additionally, the Male Classificatory Table for the Frequency Speed of Kick Test was used to evaluate athletic performance^26^.

## Methods for the metabolism experiments

### Sample collection and preparation

After venous blood samples were collected, they were centrifuged at 1100 × g for 15 min at 4 °C within 30 min. The plasma was subsequently separated and stored at − 80 °C.

### LC‒MS/MS analysis

Following slow thawing at 4 °C, an appropriate amount of the sample was mixed with a precooled methanol/acetonitrile/water mixture (2:2:1, v/v), vortexed, subjected to low-temperature ultrasonication for 30 min, and then allowed to stand at − 20 °C for 10 min. The sample was then centrifuged at 14,000 × g for 20 min at 4 °C, after which the supernatant was subsequently dried under vacuum. For mass spectrometry analysis, the dried sample was reconstituted by adding 100 μL of acetonitrile aqueous mixture (acetonitrile:water = 1:1, v/v), followed by vortexing and centrifugation at 14,000 × g for 15 min at 4 °C. The supernatant was then used for sample analysis. QC samples were prepared by extracting 10 μL from each sample, with QC analysis conducted after every 5 samples.

Mass spectrometry analysis was performed using an AB Triple TOF 6600 mass spectrometer. The ESI source conditions for post-HILIC chromatographic separation were as follows: Ion Source Gas1 (Gas1): 60, Ion Source Gas2 (Gas2): 60, Curtain gas (CUR): 30, source temperature: 600 °C, IonSapary Voltage Floating (ISVF) ± 5500 V (positive and negative modes), TOF MS scan m/z range: 60–1000 Da, product ion scan m/z range: 25–1000 Da, TOF MS scan accumulation time 0.20 s/spectra, and product ion scan accumulation time 0.05 s/spectra. The secondary mass spectrum was acquired via information-dependent acquisition (IDA) in high-sensitivity mode, with a declustering potential (DP) of ± 60 V (positive and negative modes), a collision energy of 35 ± 15 eV, and IDA settings to exclude isotopes within 4 Da. Candidate ions to monitor per cycle: 10.

### Data analysis

SPSS 25 was used to conduct the statistical tests. The distribution of variables, such as exercise intensity and performance of the experimental subjects, was assessed via the Shapiro‒Wilk test. Some data did not follow a normal distribution, leading to the expression of exercise performance data in terms of the median and interquartile range. Intergroup comparisons of sports performance were carried out via the Mann‒Whitney test for exercise intensity (a nonparametric statistical analysis), with P values less than 0.05 considered to indicate statistical significance.

Metabolites were detected via positive ion mode (POS) and negative ion mode (NEG), followed by systematic cluster analysis of the data^27^. The resulting dendrogram was calculated via average linkage, and hierarchical clustering was performed via the R package pheatmap^28^.

Principal component analysis (PCA) was conducted via the R language gModels (v2.18.1), whereas orthogonal partial least squares discriminant analysis (OPLS-DA) was performed via the R software package model.

The differentially abundant metabolite screening criteria included a variable importance for projection (VIP) of OPLS-DA ≥ 1 and a p value < 0.05 according to a single-factor t test. Fold changes (FCs) between the two groups were calculated, and a volcano plot was generated. The VIP score from OPLS-DA was used to create a chart displaying the 15 metabolites with the highest scores^29^. The Pearson correlation coefficient and p value were calculated via the R functions cor and cor.test to assess the similarity in metabolic abundance. A correlation was deemed significant when p ≤ 0.05^30^. The R corrploy package was used to produce correlation heatmaps^31^. The abundance of differentially abundant metabolites was normalized via the z score formula: z = (x—μ)/σ, where x is the specific score, μ is the mean, and σ is the standard deviation. Hierarchical clustering was performed via the R package pheatmap^28^ to create a cluster heatmap showing the cumulative difference between the two groups in terms of differentially abundant metabolites and samples.

The following method was used for Kyoto Encyclopedia of Genes and Genomes (KEGG) enrichment analysis of the differentially abundant metabolites:

$$P = 1 - \sum\limits_{i = 0}^{m - 1} {\frac{{\left( {\begin{array}{*{20}c} M \\ i \\ \end{array} } \right)\left( {\begin{array}{*{20}c} {N - M} \\ {n - i} \\ \end{array} } \right)}}{{\left( {\begin{array}{*{20}c} N \\ n \\ \end{array} } \right)}}}$$

N represents the total number of metabolites annotated by KEGG, n represents the number of differentially abundant metabolites within N, M represents the total number of metabolites annotated to a specific pathway, and m represents the number of differentially abundant metabolites within M. The p value calculated was FDR corrected, with a threshold of FDR ≤ 0.05. Pathways that met these conditions were considered to be significantly enriched in differentially abundant metabolites.

Metabolite set enrichment analysis (MSEA)^32^ was conducted via the MetaboAnalyst module and the small molecule pathway database library. Fisher’s exact test was used for overrepresentation analysis via the R package MSEAp (https://rdrr.io/github/afukushima/MSEAp/).

WGCNA^33,34^ was also performed, with a soft threshold β value of 12, a similarity of 0.8, and a minimum of 50 metabolites in each module.

## Results

### Subjects

Seventeen Taekwondo athletes participated in this experiment. The basic characteristics of the experimental subjects are shown in Table 1.

**Table 1:** Basic information of the experimental subjects and the exercise performance table.

	$\overline{X }\pm S$		Median (25%, 75% IR)		*P*_*1*_	*P*_*2*_
	20 mmHg	220 mmHg	20 mmHg	220 mmHg		
Age (Y)	19.65 $\pm$ 1.41					
Height (m)	179.06 $\pm$ 5.71					
Weight (kg)	71.41 $\pm$ 7.95					
Years of practice (Y)	7.00 $\pm$ 2.00					
BMI (kg/m^2^)	22.26 $\pm$ 2.19					
Body fat percentage (%)	13.08 $\pm$ 4.76					
FSKT_1-1_	19.7 $\pm$ 3.16	21.1 $\pm$ 1.87	20 (18,20.5)	20 (20,22)	0.021	0.031
FSKT_1-2_	19.2 $\pm$ 1.09	20.4 $\pm$ 1.41	19 (18.5,20)	20 (19.5,21.5)	0.008	0.035
FSKT_1-3_	18.7 $\pm$ 1.49	19.6 $\pm$ 1.77	19 (18,19.5)	20 (18.5,20.5)	0.021	0.058
FSKT_1-4_	18 $\pm$ 1.27	19 $\pm$ 1.90	18 (17.5,19)	19 (17.5, 20.5)	0.042	0.015
FSKT_1-5_	17.4 $\pm$ 1.66	18.7 $\pm$ 1.79	17 (17,18.5)	19 (17.5,19.5)	0.001	0.014
FSKT_total-1_	93.1 $\pm$ 6.86	98.9 $\pm$ 6.36	94 (90.5,96)	101 (94,103.5)	0.002	0.002
FSKT_KDI-1_ (%)	8.56 $\pm$ 5.93	8.91 $\pm$ 1.42			0.653	1.000
FSKT_2-1_	18.7 $\pm$ 2.44	20.1 $\pm$ 2.09	19 (18,20)	20 (18,19)	0.042	0.273
FSKT_2-2_	18 $\pm$ 1.90	19.2 $\pm$ 1.85	18 (17,19)	19 (19,20)	0.083	0.398
FSKT_2-3_	17 $\pm$ 2.15	18.6 $\pm$ 1.70	17 (16,19)	19 (18,19.5)	0.004	0.112
FSKT_2-4_	17 $\pm$ 1.70	18.6 $\pm$ 1.70	17 (16,18.5)	19 (17.5,20)	0.001	0.004
FSKT_2-5_	16.7 $\pm$ 1.96	18.1 $\pm$ 1.41	17 (16,18)	18 (17.5, 19)	0.001	0.014
FSKT_total-2_	87.4 $\pm$ 8.18	94.6 $\pm$ 8.02	87 (84.5,92.5)	96 (90.5,99.5)	0.002	0.052
FSKT_KDI-2_ (%)	9.67 $\pm$ 6.07	6.64 $\pm$ 4.02			0.055	0.119
FSKT_3-1_	18.3 $\pm$ 2.37	19.6 $\pm$ 1.70	19 (17.5,19.5)	20 (18,20.5)	0.007	0.011
FSKT_3-2_	17.1 $\pm$ 1.93	18.9 $\pm$ 1.95	18 (16,18)	19 (18,20.5)	0.002	0.039
FSKT_3-3_	16.6 $\pm$ 2.23	18.4 $\pm$ 1.84	17 (15.5,18)	19 (17,19.5)	0.004	0.046
FSKT_3-4_	16.3 $\pm$ 2.69	18.1 $\pm$ 1.60	17 (15.5, 18)	19 (16.5,19)	0.006	0.006
FSKT_3-5_	16.9 $\pm$ 1.93	18.6 $\pm$ 1.87	17 (15.5,18)	18 (17,20)	0.002	0.004
FSKT_total-3_	85.2 $\pm$ 10.05	93.6 $\pm$ 7.56	87 (80.5,90)	94 (89,99.5)	0.001	0.005
FSKT_KDI-3_ (%)	8.28 $\pm$ 6.52	6.82 $\pm$ 5.13			0.21	0.166

### Blood lactate, heart rate and RPE results at each stage

**Fig. 2:**
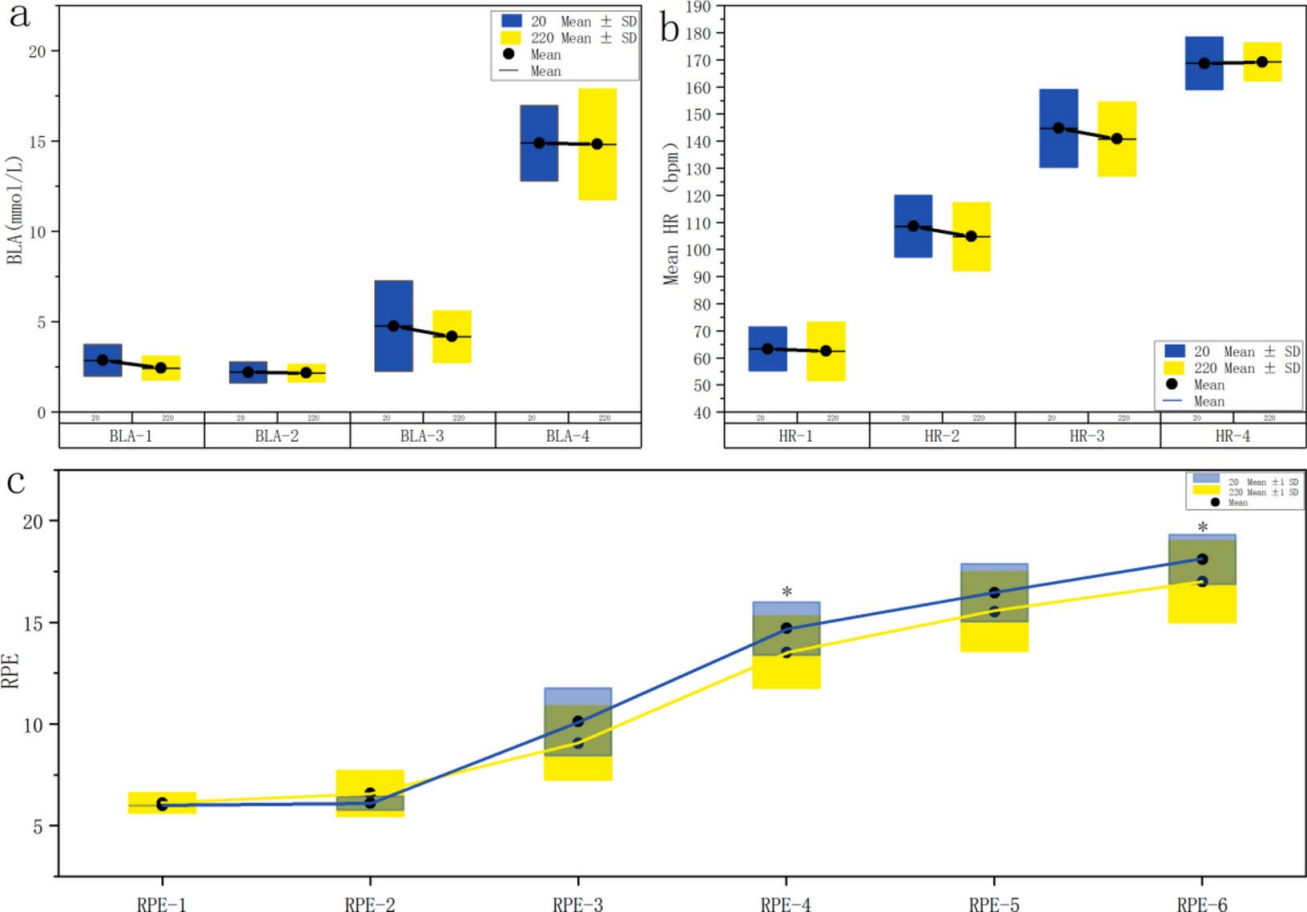
Statistical analysis of blood lactate levels, heart rate and RPE at each stage. a: BLA1-4 represent blood lactate before intervention, after intervention, after warm-up and after the exercise test, respectively. b: HR1–4 represent the average heart rate during the resting, jogging warm-up, kicking warm-up and exercise tests, respectively. c: RPE-1–3 represent the RPE before intervention, after intervention, and after warm-up, respectively. RPE-4, RPE-5, and RPE-6 indicate the RPE after the FSKT test in the different groups. * indicates a significant difference between groups (P < 0.05). The broken line shows the change in the mean RPE at each stage.

A normality test was conducted for each variable, indicating that some variables did not adhere to a normal distribution. Consequently, we employed the nonparametric paired rank sum test to examine blood lactate levels, heart rate, and the RPE. The results depicted in Fig. 2a show no significant differences in blood lactate levels between the control and experimental groups at different time points, including before and after intervention, following warm-up, and after the exercise test (P > 0.05). Similarly, there was no statistically significant difference in the average heart rate between the groups during various phases, such as the resting, jogging warm-up, kicking warm-up, and exercise test phases (P > 0.05) (Fig. 2b). However, a notable difference in the RPE was observed after FSKT between the first and third groups (P > 0.05), with the experimental group exhibiting a lower mean RPE than the control group did (Fig. 2c). The average peak heart rates during FSKT for the experimental group were 180 bpm, 184 bpm, and 187 bpm, whereas for the control group, they were 184 bpm, 185 bpm, and 185 bpm, respectively. Both groups achieved high-intensity exercise on the basis of the American College of Sports Medicine guidelines for exercise testing and prescription^35^.

### Taekwondo-specific sports performance test results

A normality test was conducted on the data, revealing that some data did not follow a normal distribution. The nonlinear paired rank sum test was subsequently used to compare sports performance between the experimental and control groups. The results revealed significant differences in the number of kicks per round of FSKT in the first group (P < 0.05). In the second group, all rounds except the second had significant differences (P < 0.05). Similarly, the third FSKT group showed significant differences in exercise performance between the experimental and control groups in each round (P < 0.05). Significant differences were also found in the total number of FSKT kicks between groups (P < 0.05), whereas no significant difference was found in the KDI of FSKT. The results, including the means, standard deviations, and P values, are presented in Table 1.

The exercise performance of the experimental subjects was assessed via the Male Classificatory Table for the Frequency Speed of Kick Test. A comparison between the control and experimental groups revealed no significant difference in the evaluation of kicking in the third round of the first FSKT group or the first, second, or third rounds of the second FSKT group. However, significant differences were found in the intergroup comparisons for the remaining rounds (P < 0.05), indicating a significant improvement in sports performance levels among the experimental subjects in the experimental group. The results, including medians, 25th and 75th percentile quartiles, and P values, are shown in Table 1.

Following notable variations in exercise performance metrics (while blood lactate levels remained consistent), a thorough examination of the metabolic profiles of participants undergoing specific tests after IPC was conducted via a metabolomic approach to explore the potential benefits of IPC in enhancing Taekwondo performance.

Table 1 FSKT_x-y_: x represents which group of FSKT, y represents which round; FSKT_total-x_: x represents which group of FSKT; IR represents the interquartile range; *P*_*1*_ and *P*_*2*_ represent the P values of the rank sum test between groups for the number of kicks and exercise level, respectively.

### Metabolite statistical results

**Fig. 3:**
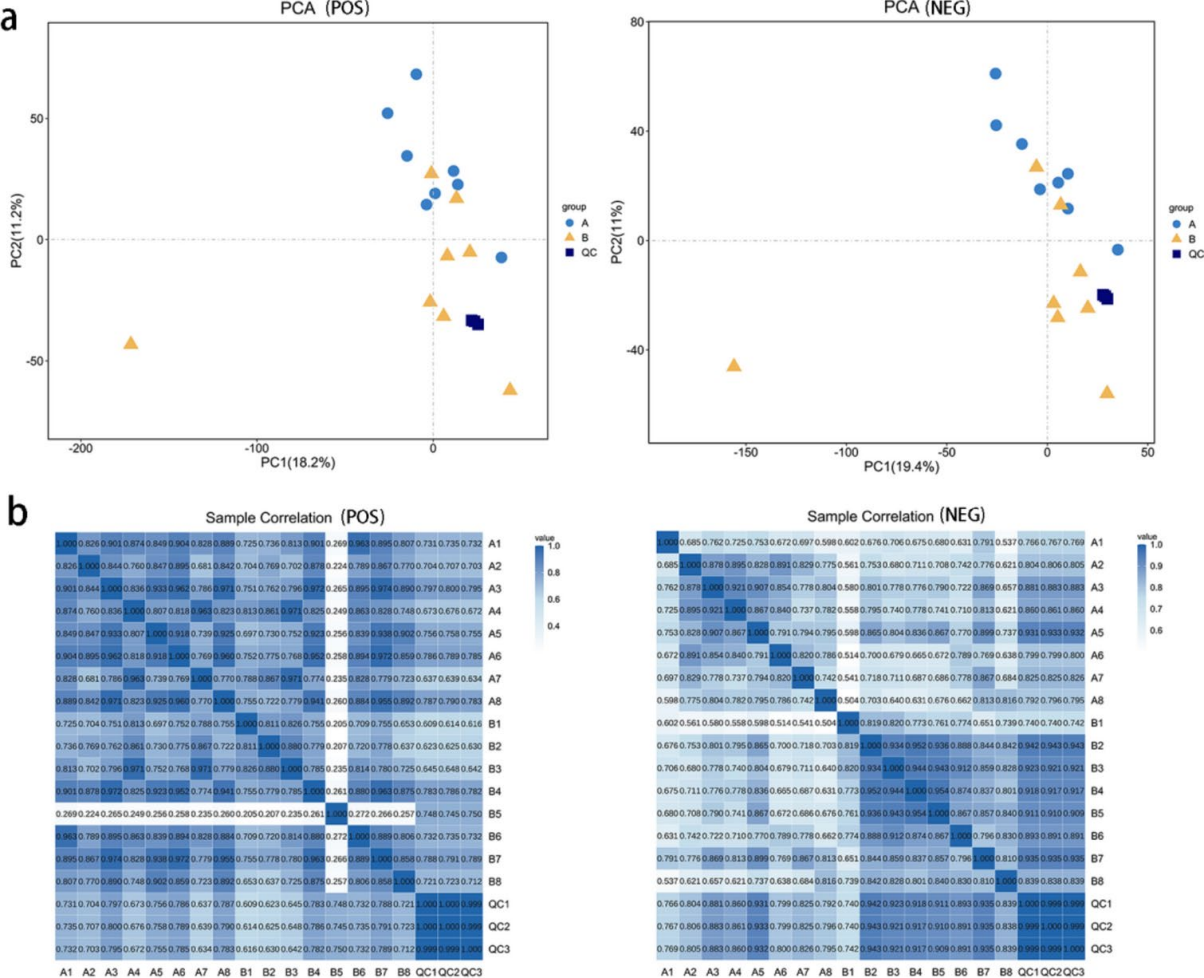
Data quality control chart. (**a**): The PC1 coordinate represents the first principal component, with the percentage in parentheses indicating its contribution to the sample difference. Similarly, the PC2 coordinate represents the second principal component, with its corresponding percentage showing the contribution to the sample difference. The colored points in the figure represent individual samples, where closer proximity indicates better repeatability within the same group. (**b**): Each row and column in the figure represents a sample, with the value in each cell representing the Pearson correlation coefficient between the two samples. A higher value and darker color indicate a stronger correlation between the samples.

Blood samples collected after IPC were assigned to Group A, whereas blood samples taken after specific exercise testing were assigned to Group B, resulting in a total of 16 samples. A total of 20,138 metabolites, comprising both positive and negative ion species, were detected in the comprehensive analysis. This data set included 2,257 known metabolites and 17,881 unknown metabolites, representing the complete range of metabolic features identified. Principal component analysis (PCA) was conducted on the metabolites from Group A, Group B, and the quality control samples, leading to the generation of positive and negative ion PCA plots (Fig. 3a). The quality control results demonstrated the high stability, excellent data quality, and reliability of the experimental data. Furthermore, a heatmap illustrating the correlations between samples in positive and negative ion modes was created on the basis of Pearson correlation coefficients (Fig. 3b). Notably, in positive ion mode, the B5 sample presented low Pearson correlation values with the other samples, indicating that it was an outlier. To ensure the accuracy of subsequent metabolomic analyses, the B5 sample was excluded.

### Identification of 109 differential metabolites through nontargeted metabolomics analysis

**Fig. 4:**
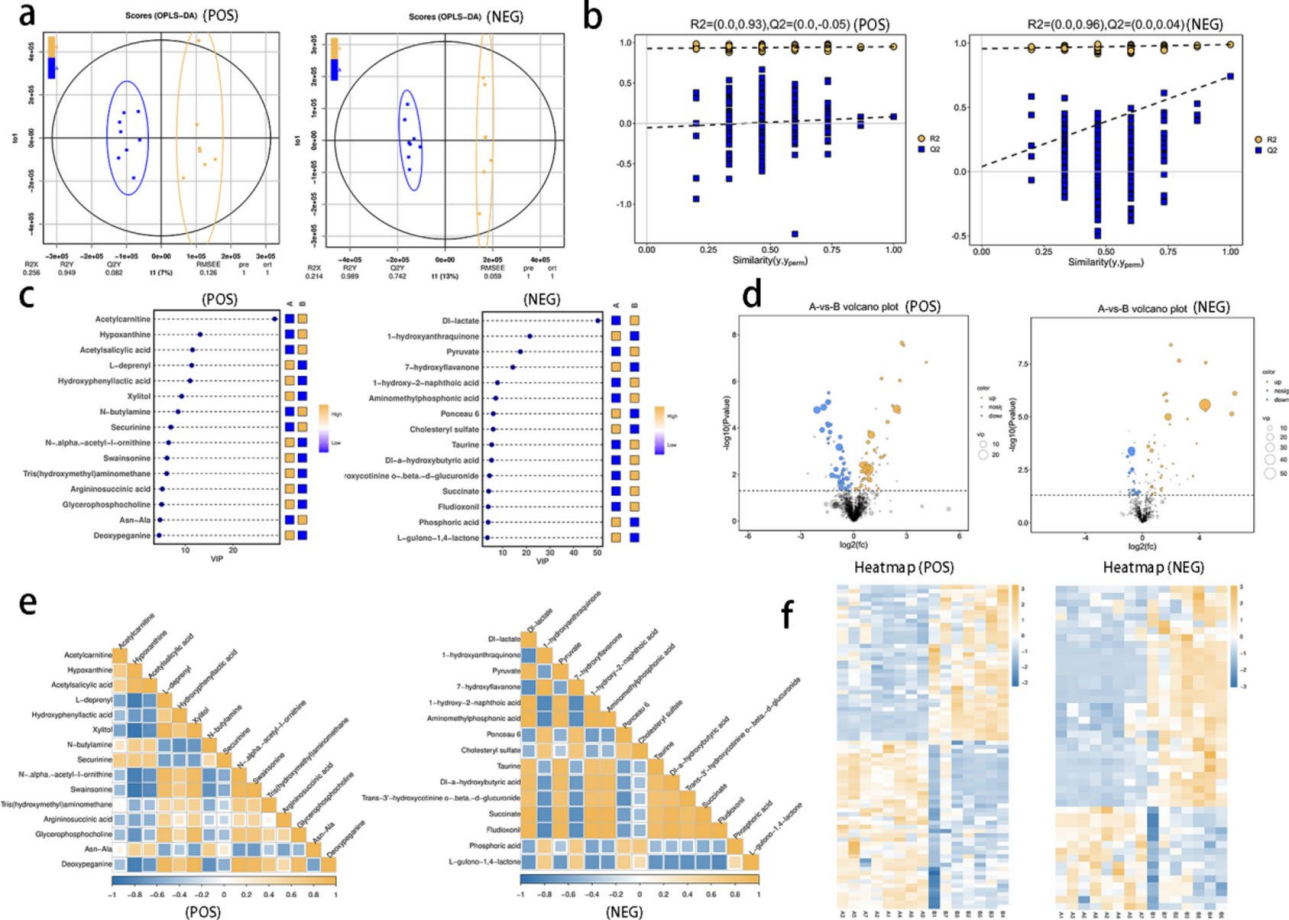
Differentially abundant metabolite screening chart. (**a**): This diagram depicts an OPLS-DA, with the blue circle representing samples from Group A and the yellow circle representing samples from Group A. Circles represent samples from group B. (**b**): If all blue Q2 points are lower than the original blue Q2 point on the right or if the intersection of the Q2 point regression line on the ordinate is less than or equal to 0, this indicates reliable model prediction results. (**c**): The VIP value is shown on the x-axis, representing the top 15 differentially abundant metabolites on the y-axis. Metabolite abundance is averaged per group and analysed via z scores, indicated by the color scale on the right. The upregulated metabolites are marked in yellow, and the downregulated metabolites are marked in blue. Metabolites with a VIP greater than 1 were considered to have significant differences, with larger VIP values indicating greater contributions to distinguishing samples. (**d**): The x-axis represents the log2-transformed fold change in metabolite abundance between comparison groups, whereas the y-axis represents the -log10-transformed P value from the T test. The vertical dashed line on the y-axis indicates the threshold for screening differentially abundant metabolites on the basis of P values. Yellow dots indicate upregulated metabolites (fold change > 1), whereas blue dots indicate downregulated metabolites (fold change < -1). Larger points correspond to higher VIP values for the metabolites. (**e**): Yellow indicates a positive correlation between changes in differentially abundant metabolites, and blue indicates a negative correlation. (**f**): Each row in the figure represents a metabolite, and each column represents a sample. The intensity of the yellow color indicates a greater abundance of the metabolite, whereas a bluer hue signifies a lower abundance.

LC‒MS/MS analysis was conducted in both positive and negative ion modes to screen for differentially abundant metabolites simultaneously. PCA was performed in both positive and negative ion modes, as shown in Fig. 3a, revealing a tendency for samples in group B to separate from those in group A along the second principal component. The OPLS-DA model was then utilized to identify the differentially abundant metabolites between groups A and B, resulting in positive and negative ion OPLS-DA score plots (Fig. 4a). A permutation test was subsequently carried out on the OPLS-DA model (Fig. 4b). The permutation test model results confirmed the reliability of the OPLS-DA model predictions for both positive and negative ions. This study highlights the notable variations in metabolite levels observed between the two sample groups. Metabolites that showed differential abundance were identified on the basis of the criteria of OPLS-DA VIP ≥ 1 and univariate t test P < 0.05. A total of 109 differentially abundant metabolites were identified, with 49 showing increased levels and 60 showing decreased levels.

The VIP values of the top 15 differentially abundant metabolites in positive and negative ion modes were calculated and used to generate the VIP statistical chart of OPLS-DA (Fig. 4c). The results indicated that Dl-lactate had the highest VIP value (VIP = 50.24), followed by acetylcarnitine (VIP = 28.69). Additionally, a volcano plot of the differentially abundant metabolites based on the fold difference (FC), VIP, and P value was created (Fig. 4d) to visualize the up- and downregulation changes in metabolite abundance. Pearson correlation coefficient analysis was conducted to examine the relationships between differentially abundant metabolites, and a heatmap illustrating the correlations among these metabolites was generated (Fig. 4e). Furthermore, Z scores were calculated for the differentially abundant metabolites, and a heatmap was generated for differentially abundant metabolite clustering (Fig. 4f), revealing notable variations in expression levels and clustering patterns among the groups.

Receiver operating characteristic (ROC) curve analysis was conducted. Among the nine differentially abundant metabolites, swainsonine, L-deprenyl, Dl-lactate, cholesteryl sulfate, 1-hydroxyanthraquinone, aminomethylphosphonic acid, Ponceau 6, taurine, and Dl-a-hydroxybutyric acid had the highest AUC values (AUC = 1). The next most common metabolites were xylitol, hypoxanthine, acetylsalicylic acid, and pyruvate, with AUC values of 0.9821 each.

### Key metabolic pathways identified through KEGG enrichment analysis of differential metabolites

**Fig. 5:**
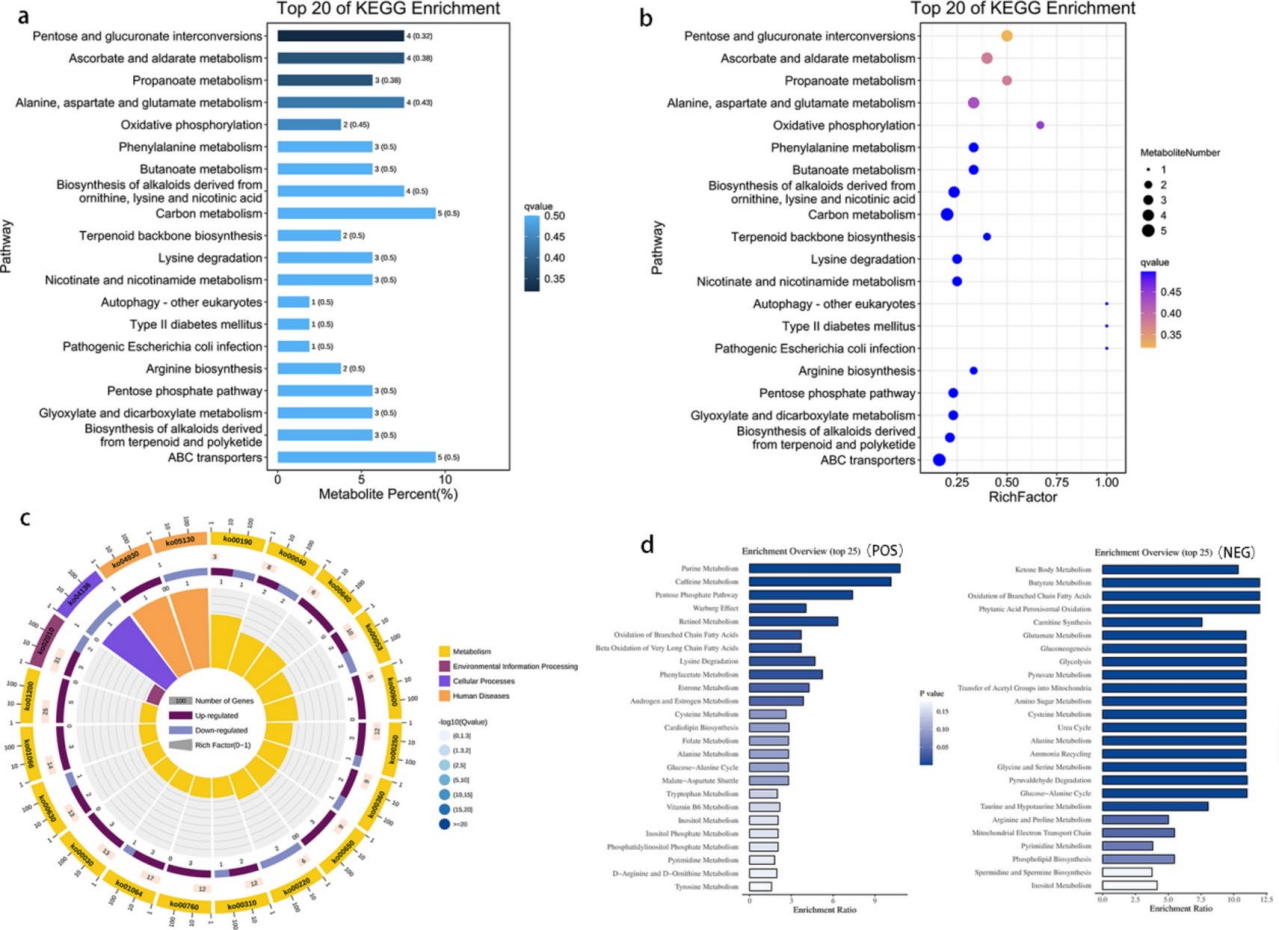
Enriched pathway analysis plot. (**a**): The KEGG enrichment bar chart was generated using the top 20 pathways with the smallest Q values. The y-axis represents the pathways, whereas the x-axis displays the percentage of each pathway compared with all the differentially abundant metabolites. Darker shades of blue in the figure indicate smaller Q values. Each bar on the chart represents the number of paths and their respective Q values. (**b**): The KEGG enrichment bubble chart displays the top 20 pathways with the smallest Q values. The y-axis represents the pathways, whereas the x-axis represents the enrichment factor (the ratio of differentially abundant metabolites in the pathway to all metabolites in the pathway). The size of each bubble corresponds to the quantity. A smaller Q value is indicated by a yellower color. (**c**): The first circle displays the top 20 enriched pathways, with the number of differentially abundant metabolites represented outside the circle. Different colors indicate different classes. In the second circle, the number of pathways and Q values are shown against the background of the differentially abundant metabolites. A longer bar indicates a greater number of differentially abundant metabolites, whereas a bluer color signifies a smaller Q value. The third circle presents a bar chart showing the proportion of up- and downregulated differentially abundant metabolites, with dark purple indicating upregulated metabolites and light purple indicating downregulated metabolites. The fourth circle displays the RichFactor value for each pathway, which is calculated as the number of differentially abundant metabolites in the pathway divided by the total quantity in the pathway. Background grid lines are included, with each grid representing 0.1. (**d**): The enrichment pathway is shown on the left side of the figure. The length of each column corresponds to the degree of enrichment, and the color represents the p value. Paths are sorted from small to large p values.

Kyoto Encyclopedia of Genes and Genomes^36^ pathway enrichment analysis was conducted on the differentially abundant metabolites, leading to the identification of 53 candidate differentially abundant metabolites with pathway annotations. Some of these metabolites include pyruvate, succinate, xylitol, D-ribulose 5-phosphate, D-arabinose, Dl-a-hydroxybutyric acid, taurine, acetylcarnitine, D-arabinonic acid, L-gulono-1,4-lactone, acetol, argininosuccinic acid, hydrocinnamic acid, deoxyadenosine, and phosphoric acid. Additionally, a total of 97 enriched pathways were identified in this study. These pathways included pentose and glucuronate interconversions (P < 0.01, involving hits such as pyruvate, D-ribulose 5-phosphate, D-arabinose, and xylitol), ascorbate and aldarate metabolism (P < 0.01, involving hits such as pyruvate, D-arabinose, D-arabinonic acid, and L-gulono-1,4-lactone), propanoate metabolism (P < 0.05, involving hits such as succinate, acetol, and Dl-a-hydroxybutyric acid), alanine, aspartate, and glutamate metabolism (P < 0.05, including hits such as pyruvate, succinate, succinic semialdehyde, and argininosuccinic acid), oxidative phosphorylation (P < 0.05, involving hits such as phosphoric acid and succinate), phenylalanine metabolism (P < 0.05, including hits such as pyruvate, succinate, and hydrocinnamic acid), and butanoate metabolism (P < 0.05, including hits such as pyruvate, succinate, and succinic semialdehyde), among others. Statistical analysis was also conducted on the KEGG enrichment pathways of the top 20 differentially abundant metabolites (Fig. 5a). A circle diagram illustrating the enrichment of the top 20 differentially abundant metabolites was subsequently generated on the basis of the P value (Fig. 5b). Furthermore, an enrichment circle plot representing the KEGG enrichment pathways of the top 20 differentially abundant metabolites was created (Fig. 5c). Notably, pentose and glucuronate interconversions presented the lowest P value among all differentially abundant metabolite KEGG enrichment pathways.

### Comprehensive metabolic pathways and potential key pathways revealed through metabolite set enrichment analysis

MSEA was conducted, with positive ion MSEA results indicating enrichment in pathways such as purine metabolism, caffeine metabolism, PPP, oxidation of branched chain fatty acids, beta oxidation of very long-chain fatty acids, lysine degradation, alanine metabolism, and the glucose‒alanine cycle, totaling 85 pathways. Negative ion MSEA revealed enrichment in 95 pathways, including ketone body metabolism, peroxisomal oxidation of phytanate, carnitine synthesis, acetyltransfer into mitochondria, butyrate metabolism, carnitine synthesis, glutamate metabolism, gluconeogenesis, glycolysis, pyruvate metabolism, alanine metabolism, and cysteine metabolism. Statistical diagrams for MSEA in positive and negative ion modes were obtained (Fig. 5d).

### Enrichment of pathways such as pentose and glucuronate interconversions revealed in WGNCA

**Fig. 6:**
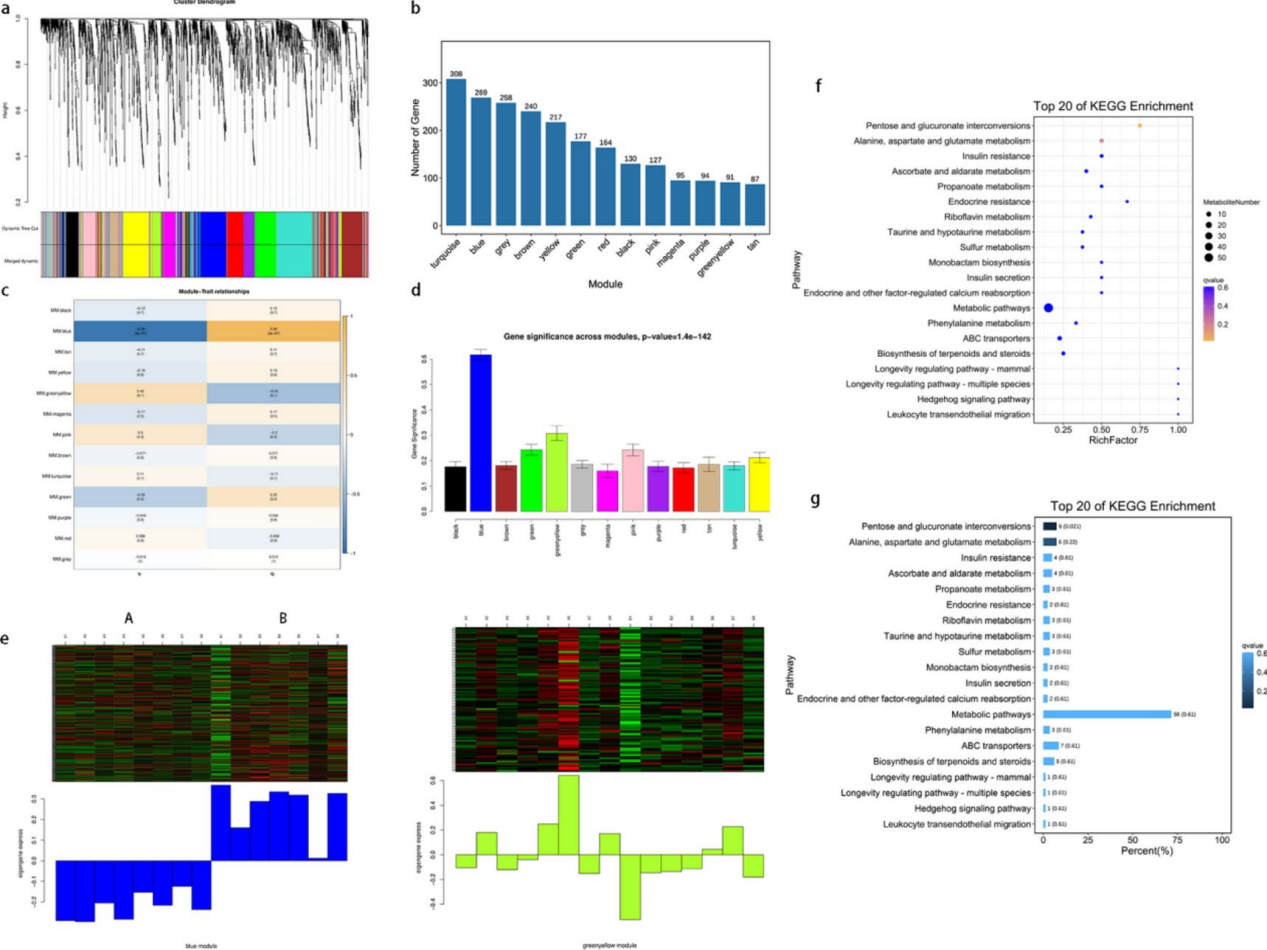
The figures of WGNCA. (**a**): Metabolites exhibiting similar expression patterns were clustered together within modules. The clustering tree branches were cut to create distinct modules, where each color represents a module and gray indicates metabolites that do not align with any specific module. (**b**): The abscissa represents each module, and the ordinate represents the number of metabolites. (**c**): The abscissa represents the trait, the ordinate represents the module, and the Pearson correlation coefficient is used for plotting. Positive correlations are depicted in yellow, whereas negative correlations are shown in blue. Darker colors signify stronger correlations. The numbers in parentheses below indicate significant P values. (**d**): Histogram of the GS of each module and trait. The abscissa is represented by different colours, and the ordinate is the GS value. (**e**): Heatmap displaying metabolite expression patterns in the blue and green‒yellow modules. The upper panel presents a heatmap displaying metabolite expression across samples within a specific module, where red denotes up-regulation and green signifies down-regulation. The lower panel depicts the module eigenvalues for various samples. (**f**): The y-axis represents various metabolic pathways, while the x-axis indicates the ratio of differentially abundant metabolites within each pathway relative to the total number of metabolites present. The size of each bubble corresponds to the quantity of metabolites, with smaller Q values resulting in a more intense yellow color. (**g**): The y-axis represents the various pathways, while the x-axis illustrates the percentage of each pathway relative to the total number of differentially abundant metabolites. Darker blue shades indicate lower Q values. The numbers displayed after the bars correspond to the number of pathways and their respective Q values.

Metabolites were subjected to WGNCA to generate a module-level clustering diagram (Fig. 6a). A total of 13 modules were identified, and a histogram displaying the number of metabolites in each module was created (Fig. 6b). We subsequently utilized module characteristic values to perform correlation analysis with the phenotypic data of groups A and B. The results revealed that the blue module presented the strongest correlation with the phenotypes of groups A and B (P < 0.001), with correlation coefficients of -0.94 and 0.94, respectively. Additionally, the green‒yellow module and the blue module displayed notable negative correlations (Fig. 6c). Furthermore, the average gene significance (GS) of metabolites within each module was calculated and is represented in a column chart (Fig. 6d), which shows that the blue module had the highest GS value, followed by the green‒yellow module. A heatmap was generated to visualize the expression of metabolites within the modules (Fig. 6e), indicating an increasing trend in the blue module and a decreasing trend in the green‒yellow module. Finally, KEGG Orthology (KO) enrichment analysis of the module metabolites revealed enrichment of pathways such as pentose and glucuronate interconversions; alanine, aspartate and glutamate metabolism; ascorbate and aldarate metabolism; and propanoate metabolism within the blue module. Pentose and glucuronate interconversions were significantly enriched (P < 0.001, Q < 0.05), and bubble and bar charts illustrating the top 20 enriched KEGG pathways in the blue module were generated (Fig. 6f). Additionally, the quantity of differentially abundant metabolites annotated by the enriched pathways was determined (Fig. 6g).

## Discussion

In this study, IPC had a significant positive effect on the sport-specific performance of Taekwondo athletes. The results of the plasma metabolomic analysis indicated that anaerobic metabolism is crucial for Taekwondo-specific tests, whereas aerobic metabolism plays a supportive role. Furthermore, the PPP and the Warburg effect are enhanced by IPC, facilitating energy replenishment. Interestingly, the differentially abundant metabolites identified not only improved the body’s resistance to oxidative stress during intense exercise but also displayed analgesic and excitatory properties that may ultimately benefit athletes’ overall performance. Notably, previous studies on high-intensity exercise metabolism have not reported functional characteristics related to improved antioxidative stress capacity and analgesic excitability.

This study presents compelling evidence that IPC enhances performance in Taekwondo athletes. The intense and frequent kicks in Taekwondo competitions rely heavily on anaerobic metabolism for energy^37^. High-level athletes tend to exhibit faster kicking speeds^38^. In this study, IPC significantly improved the total kicking performance of Taekwondo athletes in the first group of FSKTs, indicating that IPC can enhance the specific anaerobic endurance of these athletes^26^. We also observed similar characteristics in the metabolomic results. The findings revealed that the levels of dl-lactate, pyruvate, and hypoxanthine were significantly increased, with the most pronounced change observed in the level of dl-lactate. Concurrently, ROC curve analysis underscored the importance of dl-lactate in anaerobic glycolytic metabolism. Furthermore, our results revealed enrichment of the glycolysis and purine metabolism pathways in MSEA, suggesting that increased pyruvate production during high-intensity FSKT exercise accelerates lactate synthesis and ATP resynthesis^39,40^. Hypoxanthine levels reflect muscle metabolism under anaerobic conditions, and purine metabolism significantly responds to high-intensity anaerobic exercise^41^. The increase in hypoxanthine further indicates enhanced glycolysis, increased consumption and mobilization of energy substrates, and decreased ATP flux^42^. These metabolic patterns align with energy metabolism profiles typically observed during high-intensity exercise^43,44^. Additionally, research indicates that kickboxing athletes require a robust glycolytic energy supply. Therefore, we assert that IPC can significantly increase the specific anaerobic endurance of Taekwondo athletes, with glycolytic metabolism playing a crucial role in the specific test process.

As the duration of the test increased, aerobic metabolism progressively intensified^45^. Compared with the control group, the second and third FSKT groups within the experimental group demonstrated enhanced exercise performance, which may be attributed to improvements in aerobic metabolism. Among the differentially abundant metabolites, succinate and taurine, both of which are essential for energy metabolism, were significantly elevated^46,47^. KEGG enrichment analysis revealed enrichment of the TCA cycle and oxidative phosphorylation pathway, with the latter showing significant enrichment (i.e., P < 0.05, Q < 0.05). Taurine release has been shown to contribute to lipolysis, glucose uptake, and the regulation of glycolytic flux^48,49^. Succinic acid was found to increase the activity of mitochondrial complex enzymes, increase creatine kinase activity, and increase ATP production^50^. Under fast exercise conditions, succinate can aid in lactate consumption and accelerate oxidative phosphorylation through succinate receptor 1, thereby increasing energy production efficiency^51^. The activation of the oxidative phosphorylation metabolic pathway observed in our study contrasts with findings from a previous study on acute high-intensity exercise^52^. Concurrently, several studies have indicated that IPC positively influences aerobic exercise performance^9,53^. Additionally, fatty acid-derived hydrogen peroxide in peroxisomes is crucial for maintaining reduced NAD + and ATP levels in highly hypoxic environments, and there is a significant substrate-level interaction between peroxisomes and mitochondrial metabolism. This interaction, along with fatty acid breakdown, aids in the delivery of oxygen to muscles and tissues, facilitating metabolic adaptations to hypoxic conditions^54^. Taekwondo athletes may exhibit similar adaptations after IPC. Our research revealed that pathways enriched in MSEA include ketone body metabolism, branched-chain fatty acid oxidation, peroxisomal oxidation of folate esters, β-oxidation of very long-chain fatty acids, carnitine synthesis, and acetyl transport into mitochondria. These pathways contribute to improved fatty acid breakdown and oxygen transport during high-intensity exercise, leading to an increased energy supply and improved exercise performance. Importantly, however, LC‒MS/MS-based plasma metabolomics alone cannot definitively assess the specific impact of IPC on mitochondrial ATPase function and fat remodelling.

In addition to providing anaerobic and aerobic metabolic energy, our study revealed potential beneficial impacts of the PPP and the Warburg effect on energy metabolism following high-intensity exercise post-IPC. The pentose phosphate pathway, a glucose oxidation pathway that runs parallel to upper glycolysis^55^, primarily involves the production of nicotinamide adenine nucleotide phosphate (NADPH) and ribose 5-phosphate (R5P) while releasing energy^56–58^. Interestingly, our KEGG enrichment analysis of differentially abundant metabolites and MSEA highlighted the enrichment of the PPP, a pathway that has rarely been discussed in previous exercise metabolomics studies. These findings suggest that PPP activation due to IPC may influence the energy metabolism of Taekwondo athletes during subsequent specialized tests. Furthermore, we observed enrichment of the Warburg effect pathway via MSEA. Some studies suggest that the body enhances the Warburg effect (aerobic glycolysis) to increase bioenergetic ATP circulation in high metabolic states, despite less efficient glucose consumption. Additionally, transient ischemic conditions may also trigger the activation of the Warburg effect pathway^59^.

Further investigation revealed certain differentially abundant metabolites and enriched pathways with functional characteristics that may improve the body’s capacity to resist oxidative stress. High-intensity training is known to disrupt the redox balance of skeletal muscle^60^, leading to oxidative stress and subsequent muscle fatigue^61,62^. However, in this study, despite rigorous testing, several differentially abundant metabolites, such as 7-hydroxyflavanone, acetyl-L-carnitine, and acetylsalicylic acid, were notably upregulated. These upregulated metabolites have antioxidant properties that can increase the body’s resistance to oxidative stress and maintain redox balance during high-intensity exercise^63–65^. Moreover, the levels of argininosuccinic acid and tetradinone notably decreased. Argininosuccinic acid diminishes antioxidant defenses in the cerebral cortex, induces oxidative stress in the striatum, and elevates reactive oxygen species in the cerebral cortex^66^. Moreover, a decrease in tetradinone levels hinders mutations and safeguards the antioxidant system from harm^67,68^. A decrease in these metabolites alleviates oxidative stress and contributes to maintaining redox balance. According to the KEGG enrichment analysis of the differentially abundant metabolites and WGCNA, the ascorbate and aldarate metabolism pathways were enriched. These findings indicate that these pathways were activated after high-intensity special testing following IPC. Ascorbate and aldarate metabolism are vital carbohydrate pathways that safeguard cells from oxidative damage^69^. Previous studies have demonstrated that IPC can increase the body’s antioxidant capacity and mitigate inflammatory responses^70^. These results suggest that after performing three sets of FSKTs following IPC, Taekwondo athletes may still maintain a relatively good and stable redox state, delaying muscle fatigue and enabling higher output during exercise, ultimately leading to improved sports performance.

In addition, we found that acetylsalicylic acid and caffeine were unexpectedly significantly upregulated. Acetylsalicylic acid can increase pain tolerance, reduce pain and injury-induced inflammation, and improve endurance and neuromuscular performance^71,72^. Caffeine acts on the central nervous system by blocking adenosine receptors, leading to increased neurotransmitter release, motor unit firing rates, and pain relief^73,74^. It also increases dopamine levels in brain regions associated with attention and enhances sodium/potassium pump activity for muscle contraction^75^. Previous studies have demonstrated that IPC reduces pain sensitivity during painful cold stimulation, which aligns with our findings^76^. Therefore, the increased endogenous production of acetylsalicylic acid and caffeine might enhance the athletic performance of taekwondo athletes post-IPC because of their central analgesic and stimulant effects. This could explain why the experimental group showed improved performance during intense exercise while reporting reduced subjective fatigue.

## Conclusion

IPC significantly enhances the specific sports performance of taekwondo athletes. Plasma metabolomics revealed the upregulation of Dl-lactate, pyruvate, and hypoxanthine in the athletes’ bodies, along with the enrichment of the glycolysis and purine metabolism pathways, suggesting that anaerobic metabolism plays a crucial role in FSKT. Additionally, the upregulation of succinic acid and taurine, along with the enrichment of the TCA cycle and oxidative phosphorylation pathways, suggested a supporting role for aerobic metabolism. The PPP and the Warburg effect may also provide supplementary energy for high-intensity exercise. Notably, after IPC and high-intensity exercise, the expression of 7-hydroxyflavanone, acetyl-L-carnitine, acetylsalicylic acid, and caffeine increased, whereas the expression of argininosuccinic acid and tetradinone decreased, leading to significant enrichment of ascorbate and aldarate metabolism. These metabolic alterations may enhance the body’s ability to combat oxidative stress during exercise, improve muscle fatigue resistance, and provide analgesic and stimulating effects, thereby enhancing exercise performance.

However, this study has certain limitations, as it focused only on metabolic changes in the experimental group without a non-IPC metabolic group as a control. This limits our ability to fully comprehend the specific impact and magnitude of IPC on the Taekwondo exercise. Additionally, owing to limited research methods, the dissolved O_2_ in blood and deutenomics arguments were not determined, preventing the assessment of IPC’s effect on mitochondrial ATPase functions and Acyl- and acetyl-carnitine profiles. Future research will focus on targeted testing based on mechanisms identified through metabolomics to elucidate how IPC enhances sports performance.

## Data Availability

The complete list of metabolites with their MS/MS identification and raw data sets in this study can be found in the online repositories. (https://doi.org/10.6084/m9.figshare.27024757.v3).

## Abbreviations

IPC: Ischemic preconditioning
OPLS-DA: Orthogonal partial least squares discriminant analysis
KEGG: Kyoto encyclopedia of genes and genomes
MSEA: Metabolic set enrichment analysis
WGNCA: Weighted gene coexpression network analysis
FSKT: Frequency speed of the kick test
FDR: False discovery rate
PPP: Pentose phosphate pathway
mK_ATP_: Selective activation of mitochondrial ATP-sensitive potassium channels
RPE: Rating of perceived exertion
BP: Blood pressure
POS: Positive ion mode
NEG: Negative ion mode
VIP: Variable importance for the projection
ROC: Receiver operating characteristic
PCA: Principal component analysis
QC: Quality control
LC‒MS/MS: Liquid chromatography‒tandem mass spectrometry
AUC: Area under the curve
